# Erector Spinae Plane Block as an Anesthetic Technique for Open Gastrostomy: A Case Report

**DOI:** 10.7759/cureus.76799

**Published:** 2025-01-02

**Authors:** Tiago Miguel Cardoso, Catarina Viegas, Erica Amaral, Miguel Sá, Rita Torgal, Susana Caramelo

**Affiliations:** 1 Anesthesiology, Intensive Care and Emergency Department, Unidade Local de Saúde de Santo António, Porto, PRT; 2 Anesthesiology Department, Unidade Local de Saúde de Trás-os-Montes e Alto Douro, Vila Real, PRT

**Keywords:** anesthetic management, bronchoesophageal fistula, erector spinae plane block, open gastrostomy, regional anesthesia

## Abstract

The erector spinae plane block (ESPB) is a relatively new technique that has been gaining attention for its versatility in providing thoracoabdominal postoperative analgesia. This case report describes the successful use of a bilateral ESPB with sedation as an anesthetic technique in a 50-year-old male diagnosed with esophageal cancer with a suspected bronchoesophageal fistula, who required an open gastrostomy. Given the patient's condition and the potential high risk of respiratory complications associated with general anesthesia, ESPB was chosen for its potential ability to offer effective surgical anesthesia and postoperative analgesia with minimal risks. A total of 12 mL of ropivacaine 0.5% was administered on each side at T9 level, achieving an effective sensory block from T7 to T10. Throughout the procedure, the patient maintained spontaneous ventilation and experienced no intraoperative complications. This report highlights the potential role of ESPB in high-risk patients. Further research is required to validate its efficacy and safety as an anesthetic technique for minor surgical procedures.

## Introduction

The erector spinae plane block (ESPB) is a relatively recent advancement in regional anesthesia. Introduced by Forero et al. in 2016, this technique was originally described for the treatment of chronic thoracic neuropathic pain and postoperative pain in thoracic surgery. It involves the injection of local anesthetic into the fascial plane located deep in the erector spinae muscle group and superficial to the transverse processes of the vertebrae [1]. Unlike traditional nerve blocks, which target specific peripheral nerves, ESPB relies on the spread of local anesthetic within a specific fascial plane to achieve a multi-dermatomal sensory block [2].

ESPB has gained significant attention In the past few years, with several publications highlighting its efficacy as a postoperative pain management technique [3,4]. Furthermore, this block has also been successfully described as a sole anesthetic modality for some selected surgical procedures, expanding its potential application beyond postoperative pain management. In this report, we discuss the use of a bilateral ESPB with sedation as an anesthetic technique, in a 50-year-old male with esophageal cancer and a suspected bronchoesophageal fistula, who required an open gastrostomy.

## Case presentation

**Figure 1:**
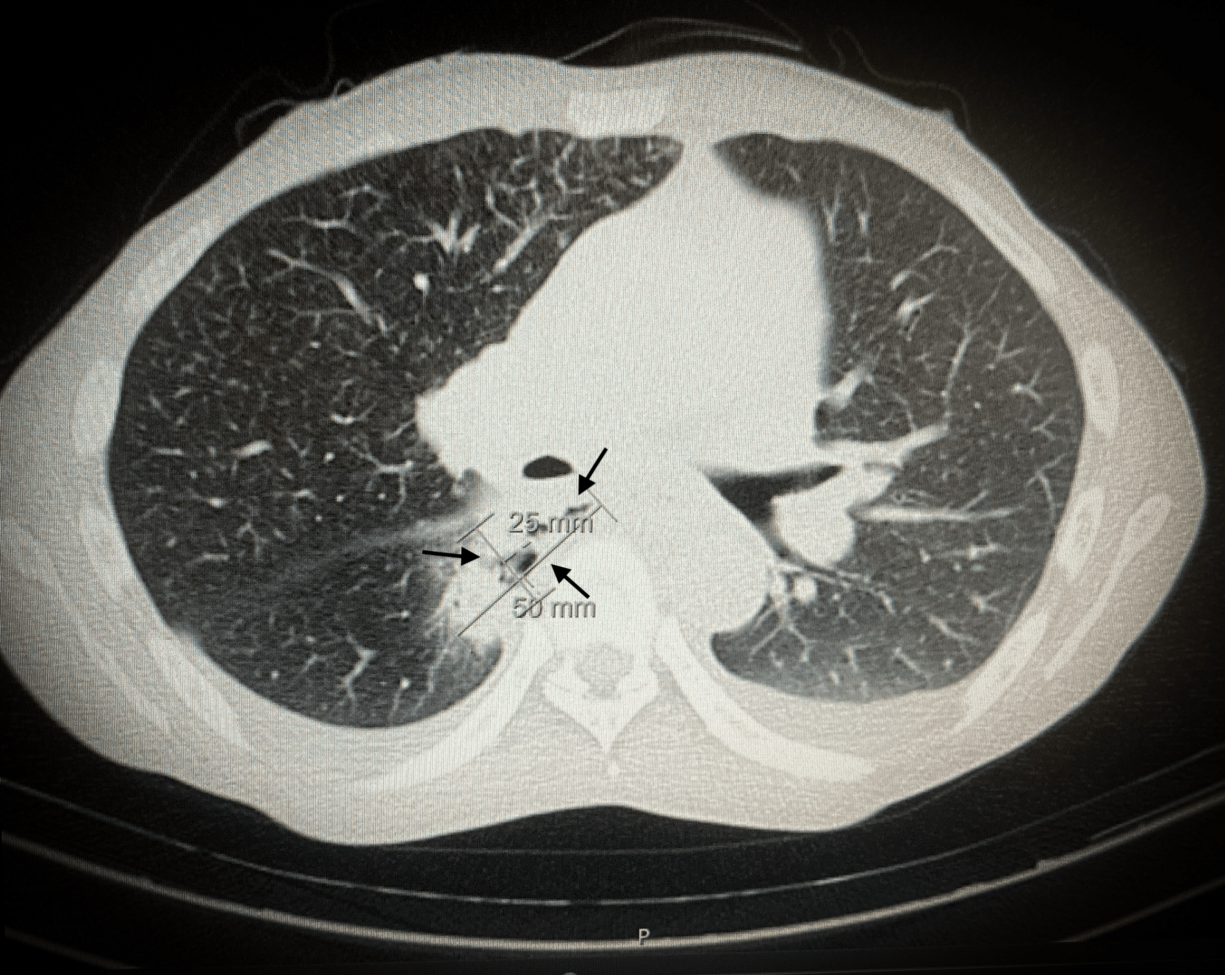
CT scan showing a fistulous tract extending towards the right-sided paravertebral collection containing both fluid and air (arrows) CT: computed tomography

The patient was a 50-year-old male, American Society of Anesthesiologists (ASA) grade III, weighing 42 Kg and 160 cm tall (BMI: 16.4 Kg/m^2^) with a history of dysphagia, anorexia, vomiting, and weight loss of 10 Kg in the last three months and was diagnosed with esophageal cancer. The CT scan showed marked wall thickening and heterogeneous attenuation of the mid-esophageal segment, highly raising concerns for a neoplastic process. Additionally, a focal disruption was observed, with a fistulous tract extending towards the right-sided paravertebral collection containing both fluid and air (Figure 1).

**Figure 2:**
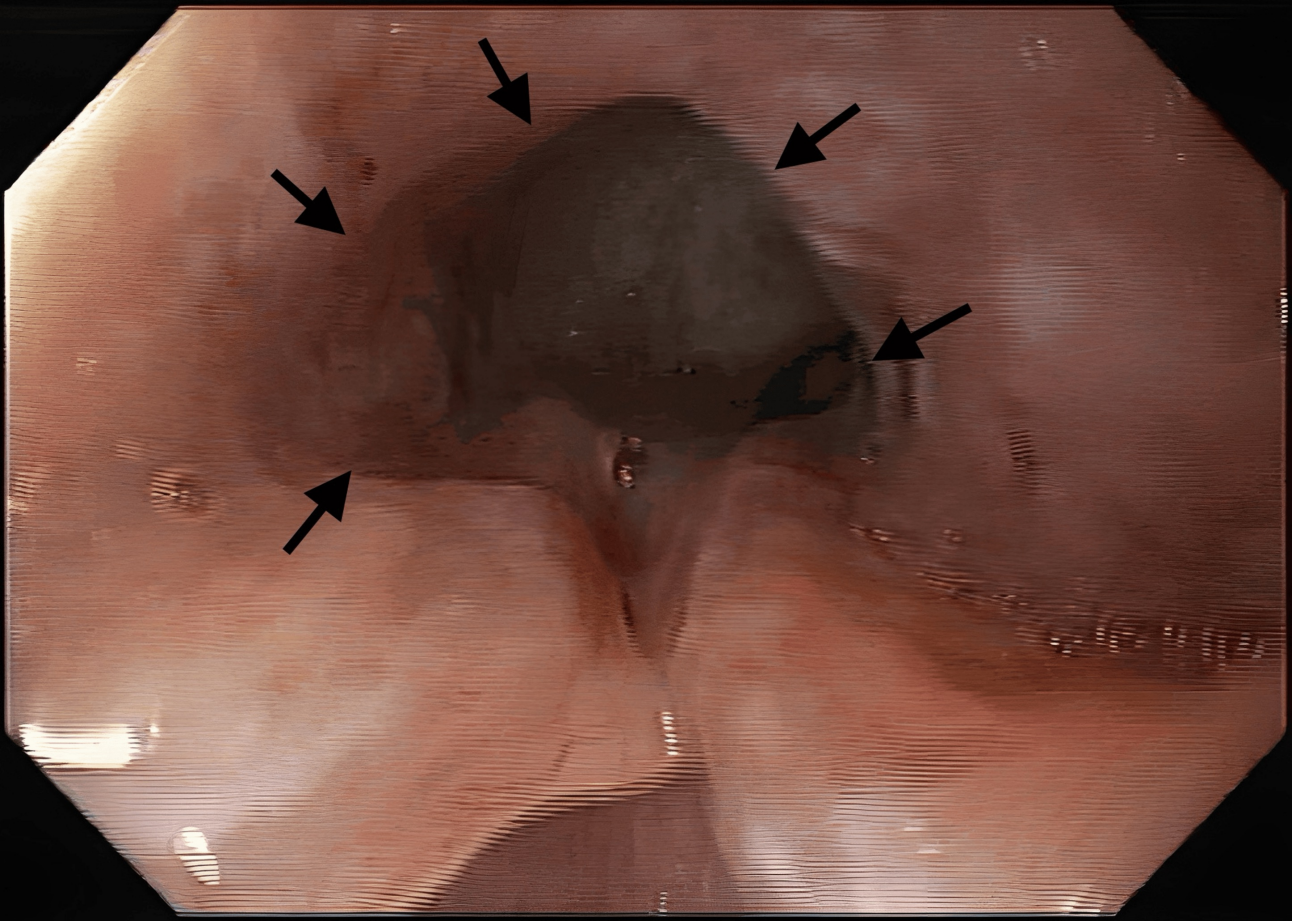
Upper endoscopy showing an ulcerated tumor occupying approximately two-thirds of the esophageal lumen (arrows)

Upon upper endoscopic examination, an ulcerated tumor was observed occupying approximately two-thirds of the esophageal lumen, which precluded further advancement of the endoscope (Figure 2). Additionally, attempts at air or water insufflation during the procedure induced coughing reflexes, suggesting a potential fistula between the esophagus and the respiratory tract. These findings were corroborated by the CT scan results, which indicated a fistulous tract extending from the esophagus to a paravertebral collection. The flexible bronchoscopy examination revealed a slight stenosis of the B6 segment, where air bubbles were observed, without the identification of a clear drainage solution.

Given the patient’s severe clinical presentation, an open gastrostomy was proposed. Due to the high risk of respiratory complications associated with general anesthesia, particularly in a patient with a potential bronchoesophageal fistula, a less invasive approach was sought and bilateral ultrasound-guided ESPB with sedation was chosen as the sole anesthetic technique.

Anesthetic management

The patient was monitored as per ASA standards and positioned in right lateral decubitus. A high-frequency linear ultrasound probe was positioned in a longitudinal orientation to obtain a parasagittal view and slowly moved laterally until the transverse process was visible as a flattened hyperechoic structure. The ESPB was performed at the T9 level with a 22G x 50 mm needle (Pajunk SonoPlex II®) using a caudocranial in-plane needling approach under sterile conditions. The bevel of the needle was pointed posteriorly and superiorly and advanced under ultrasound guidance towards the posteroinferior corner of the transverse process. Once the needle tip was below the erector spinae muscle, the hydrodissection of a small bolus of 2 mL of saline solution confirmed the correct position, visualizing the erector spinae muscle separating from the transverse process. A total of 12 mL of ropivacaine 0.5% was injected on each side, taking care to keep it within the safe dosage limits. After 30 minutes, pinprick and temperature sensations were tested and showed a sensory blockade from T7 to T10.

**Figure 3:**
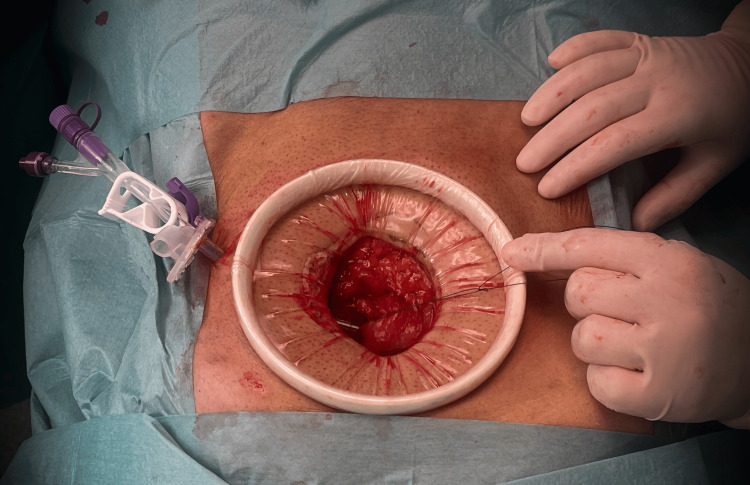
Open gastrostomy through a supraumbilical midline incision

Sedation was then performed with target-controlled infusion (TCI) of propofol based on the Schnider model with a target concentration effect of 2.5 mcg/ml and 10 mg bolus of ketamine. A supraumbilical midline incision was performed; the abdominal cavity was cautiously accessed, and a gastrostomy was made with a 22 F feeding tube (Figure 3).

The surgical procedure was completed in approximately 45 minutes. Throughout the procedure, the patient maintained spontaneous ventilation, remained hemodynamically stable, and no intraoperative complications were encountered. After the surgery, the patient received intravenous paracetamol 1000 mg and ketorolac 15 mg as part of a multimodal postoperative analgesia regimen, and no opioids were used. The patient's postoperative recovery was uneventful.

## Discussion

Several publications in the current literature have highlighted the role of ESPB as an effective regional anesthetic technique for a wide range of surgical procedures, including thoracic, breast, abdominal, gynecological, neck, and even hip surgeries [3]. With surgical techniques becoming less invasive, the introduction of enhanced recovery after surgery, and the increased use of anticoagulation therapies, the use of neuraxial anesthesia has decreased. ESPB is considered a potential alternative analgesic option to thoracic epidural analgesia and paravertebral blocks, especially in cases where these techniques are contraindicated. ESPB has a good safety profile with very few reported complications and, perhaps, its greatest strength is its simplicity with a wide range of clinical applications [2,4,5].

A perceived key benefit of the ESPB over other fascial blocks for abdominal procedures (like the rectus sheath block or transversus abdominis plane block) is the anterior spread of injectate into the paravertebral and epidural space. This would block not only spinal nerve roots but also rami communicantes transmitting sympathetic fibers, leading to relief from visceral pain. Thus, the ESPB has the potential to provide both somatic and visceral sensory blockade, which would make it a useful regional anesthetic technique for abdominal surgery [6,7].

While ESPB is primarily used as a postoperative analgesic technique, its use as the sole anesthetic modality for certain minor surgical procedures has been described [3,8,9]. A case series of 11 patients undergoing abdominal surgery with ESPB reported that two patients did not require general anesthesia [4]. In another case, Tulgar et al. [10] reported the successful use of ESPB for ileostomy closure in a patient in whom the risks associated with general anesthesia were considered significant. Bagaphou et al. [3] have also described a case of an open gastrostomy performed successfully with an ESPB as a sole anesthetic technique; however, its application for these procedures has not yet been widely recognized. A recent case report by Viswanath et al. [11] described the successful use of thoracic epidural anesthesia in an open gastrostomy. While this technique is a suitable option, it may pose increased technical difficulty and have a potential impact on hemodynamic stability.

Our patient had a high risk of respiratory complications associated with general anesthesia, primarily due to the presence of a suspected bronchoesophageal fistula. Bearing in mind the anesthetic plan, ESPB was considered a suitable option. The transverse processes, which serve as clear ultrasound landmarks, can be easily visualized, allowing for precise needle placement. Additionally, the injection site is located at a safe distance from the neuraxis, pleura, and major vascular structures, minimizing the risk of complications associated with inadvertent nerve or vascular puncture [4,12]. The patient successfully underwent open gastrostomy using ESPB combined with sedation as a sole anesthetic technique, while maintaining spontaneous breathing.

In our patient, the ESPB was performed at T9 level using a total dose of 12 mL of ropivacaine 0.5% on each side, with an apparent diffusion of local anesthetic from T7 up to T10. The dosage of local anesthetic was adjusted based on the patient's weight with a focus on minimizing potential side effects. Epinephrine supplementation could also be added to potentially increase the volume of local anesthetic while reducing its systemic absorption. The literature remains inconsistent regarding the optimal volume of local anesthetic required to achieve adequate coverage of a specific dermatome. A review by De Cassai and Tonetti [13] involving 14 studies found a wide range of volumes (from 2.5 mL to 6.6 mL), with a median value of 3.4 mL needed to cover a single dermatome. Chin and El-Boghdadly [7] also state that the physical spread is volume-dependent, and in adult patients, the optimal volume should be greater than 10 mL, with 20-30 mL being most used, taking into account that the targeted transverse process should be congruent with the spinal nerves innervating the area of the desired effect. Moreover, the spread into the paravertebral space is most likely accomplished at the level of injection as well as one to two levels higher and lower.

It is also important to acknowledge that the efficacy of the ESPB can vary depending on the vertebral level. In the thoracic region, the dorsal rami divide into lateral and medial branches, which innervate the thoracic segments. In contrast, in the lumbosacral region, each dorsal ramus splits into medial, intermediate, and lateral branches, which merge with branches from other levels to innervate the waist, lumbar spine, and gluteal regions. These anatomical variations can influence the spread of local anesthetic and the effectiveness of the block [5].

ESPB has emerged as a promising technique and studies have shown that a bilateral block at the T7-T9 level can be an effective component of a multimodal analgesic strategy for abdominal surgery [14,15]. Publications relating to the ESPB have witnessed exponential growth in the field of regional anesthesia, along with the rapid expansion of potential indications for its use. However, to the best of our knowledge, no comparative studies evaluating the efficacy of ESPB as an anesthetic technique relative to neuraxial or paravertebral blockade have been performed. The unclear mechanism of action, variable sensory block, and limited robust evidence make it a difficult block to consider as a sole anesthetic technique. Additionally, publication bias may influence the available evidence, as negative or inconclusive studies are less likely to be reported [5].

While ESPB has shown great promise as a regional anesthetic technique for some minor surgeries, further research is needed to validate these findings, and randomized controlled trials are essential to establish its true efficacy and safety for specific surgical procedures, such as open gastrostomy.

## Conclusions

ESPB represents a remarkable advancement in regional anesthesia, offering a versatile and effective alternative for both multimodal postoperative pain management and as an anesthesia technique, particularly in high-risk patients. The utility of ESPB in some minor surgical procedures, combined with its favorable safety profile and the potential for carrying out multimodal analgesia, showcases its growing role in anesthesia practice. Due to the inherent limitations of case reports, which limit drawing broad conclusions, continued research is essential to elucidate the method's mechanisms of action and to assess its effectiveness, particularly when used as an anesthetic technique.
